# A Fine Analysis of Zn Species Structure and Distribution in Zn/ZSM-5 Catalysts by Linear Combination Fitting Analysis of XANES Spectra

**DOI:** 10.3390/molecules29030631

**Published:** 2024-01-29

**Authors:** Baichao Li, Jie Gao, Jiabei Shao, Rui Geng, Zhangfeng Qin, Jianguo Wang, Weibin Fan, Mei Dong

**Affiliations:** 1 State Key Laboratory of Coal Conversion, Institute of Coal Chemistry, Chinese Academy of Sciences, Taiyuan 030001, China; 2 University of Chinese Academy of Sciences, Beijing 100049, China; 3 College of Chemistry and Materials, Taiyuan Normal University, Jinzhong 030619, China

**Keywords:** Zn-containing ZSM-5, aromatization, XANES, LCF, active site, dehydrogenation

## Abstract

Investigating the distribution of different Zn species on Zn-containing zeolite catalysts is crucial for identifying the active sites and establishing the relationship between the catalyst’s structure and its activity in the process of ethylene aromatization. By utilizing X-ray absorption near edge spectra (XANES) of various reference samples, this study employed linear combination fitting (LCF) analysis on XANES spectra of real samples to accurately measure the changes in the distribution of Zn species in Zn-containing HZSM-5 zeolites under different Zn sources and loadings. The results showed that ZnOH^+^, ZnO clusters, and ZnO crystalline structures coexist in Zn/HZSM-5 catalysts prepared through physical mixing and incipient wet impregnation methods. A similar trend was observed for catalysts prepared using different methods, with an increase in Zn content resulting in a decrease in the proportion of ZnOH^+^ and a significant increase in the amount of larger ZnO crystals. Furthermore, ZnO clusters were confined within the zeolite pores. The findings of this study established a direct correlation between the amount of ZnOH^+^ determined through LCF analysis and both the rate of hydrogen production and the rate of aromatics formation, providing strong evidence for the catalytic role of ZnOH^+^ as an active center for dehydrogenation, which plays a key role in promoting the formation of aromatics. The method of LCF analysis on XANES spectra allows for the determination of the local structure of Zn species, facilitating a more precise analysis based on the distribution of these species. This method not only provides detailed information about the Zn species but also enhances the accuracy of the overall analysis.

## 1. Introduction

Aromatic hydrocarbons, such as benzene, toluene, and xylenes, are commonly used as raw materials for the production of high-value-added products like plastics, paints, cosmetics, and pharmaceuticals. These aromatic hydrocarbons are primarily obtained from naphtha through industrial processes like catalytic reforming or steam cracking. However, due to the increasing global demand for aromatics and the limited availability of oil resources, it is essential to develop alternative processes for the production of BTX from non-petroleum sources. As a result, there has been widespread attention to the direct conversion of methanol into aromatics (MTA) using zeolitic catalysts in recent years [1,2].

ZSM-5 zeolites are widely used as acid catalysts in the aromatization reaction because of their large surface area, high thermal/hydrothermal stability, unique pore structure, and shape selectivity [3,4,5]. Various reactions, such as oligomerization, cracking, dehydrogenation, and cyclization, can occur simultaneously on ZSM-5 zeolites during the aromatization process. By altering the preparation of ZSM-5, such as the Si/Al ratio [6,7], crystal structure, and morphology [8,9,10], as well as the source of silicon or aluminum [11,12], the selectivity of product formation can be improved to some extent. Even so, aromatic selectivity is still limited by cracking side reactions on ZSM-5 zeolites. To improve the catalytic performance of ethylene aromatization, various metal species, including Zn [13,14,15], Ga [16,17], Pt [18,19], etc., are used to modify ZSM-5. This is done by adjusting the acid properties and introducing different active species. Zn species, which possesses excellent dehydrogenation catalytic behaviors, are often introduced for modification to enhance the selectivity of ZSM-5 for aromatics.

The aromatization performance of Zn-containing HZSM-5 is strongly influenced by the structure of Zn species (coordination environment and existence states), which in turn depends on factors such as the preparation method [20,21,22], the amount of Zn introduced into the ZSM-5 zeolites [23], and the post-treatment conditions [24]. A lot of efforts have been made in researching the state of Zn species. Kolyagin et al. [25] studied the state of Zn species in HZSM-5 zeolites prepared by the impregnation method and found that there are three types of Zn species, namely, ZnO macro-crystalline, ZnO clusters with a diameter around 10 Å, and independent Zn ions (Zn^2+^ and ZnOH^+^). Berndt et al. [26,27] studied Zn species by temperature-programmed surface reaction using CO as a probe (CO-TPSR) and confirmed the existence of ZnOH^+^ species. Chen et al. [28] conducted a study on Zn-containing HZSM-5 from different preparation methods and observed that the primary Zn species in samples prepared by physical mixing were ZnO macro-crystalline, with a small quantity of ZnOH^+^ species. On the other hand, samples prepared by the ion exchange method contained ZnOH^+^ species and ZnO clusters dispersed in the zeolite pore channels. Moreover, the ZnOH^+^ species is considered to be the active species in aromatization reaction [29,30,31,32]. Niu et al. [33] investigated the states and content of Zn species during the process of MTA on Zn-containing HZSM-5catalyst and found a linear positive correlation between the aromatic selectivity and the content of ZnOH^+^ species. Furthermore, the relative proportion of each Zn species is also affected by multiple factors including the content of zinc sources introduced into zeolite, preparation method, and atmosphere. Substantial studies have concluded that there is a synergistic effect between metallic species and Brönsted acid on HZSM-5 [34,35], whereas the state and role of Zn species as well as the interaction between Zn species and the protonated acidic sites are still rather debatable.

Currently, multiple characterization techniques, such as XRD, Raman, UV-vis, XPS, and NMR, have been employed to obtain the information of Zn species in zeolite [36]. However, these techniques do not fully provide information on the ZnO clusters confined within the channels or the highly dispersed ZnOH^+^ species. The XAFS technique, which employs EXAFS to analyze the chemical environment, bond distances, and coordination number, as well as XANES to study the local structure and evolution of metal ions, is an effective method for analyzing these aspects [37]. Geng et al. [38,39] used linear combination fitting (LCF) analysis in XANES to study the composition and evolution of Zn species at different stages of ethylene aromatization reaction, providing a possibility for quantitative analysis of Zn species.

In this paper, XANES technology, especially the LCF analysis method, combined with EXAFS and conventional characterization methods (XRD, N_2_ physical adsorption, ICP, and UV-vis), was used to quantitively investigate the state of Zn species in catalysts prepared with different methods and Zn contents. The prepared catalysts were evaluated for the ethylene aromatization reaction and the relationship between the structure of Zn species, and the aromatization activity was elucidated accordingly.

## 2. Results and Discussion

### 2.1. Textural and Physical Properties of Zeolite Catalysts

**Figure 1:**
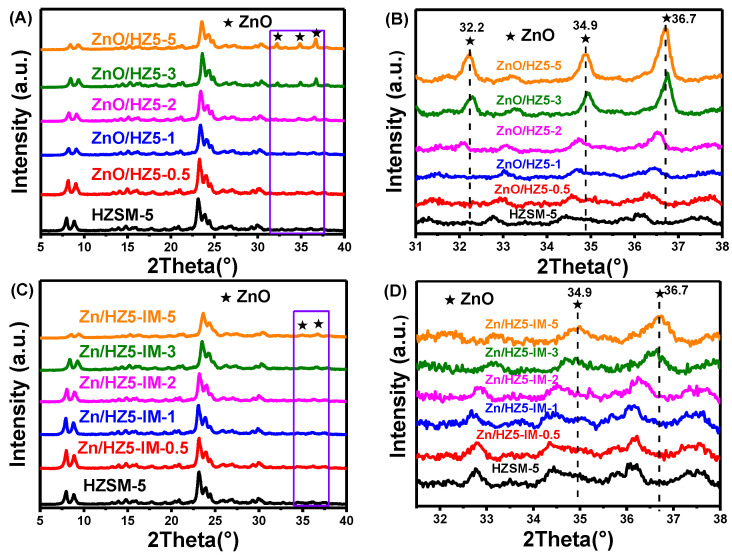
XRD patterns (**A**) and locally enlarged XRD patterns (**B**) of ZnO/HZ5−*x* samples, XRD patterns (**C**), and locally enlarged XRD patterns (**D**) of Zn/HZ5-IM−*x* samples.

The textural and physical properties, such as crystallinity, surface areas, and pore volumes, of the Zn-containing catalysts are presented in Table 1. The data indicate that the introduction of Zn species led to a decrease in catalyst crystallinity, particularly when the Zn content surpassed 2 wt.%. Concurrently, there was a reduction in both the specific surface area and pore volume of the catalyst, which was caused by the blockage of some pores after the introduction of Zn. Figure 1A,B presents the XRD patterns of ZnO/HZ5−*x* samples and Zn/HZ5-IM−*x* samples with different Zn contents prepared via physical mixing and impregnation methods, respectively. Interestingly, the characteristic diffraction peak of ZnO (2θ = 32.3°, 34.9°, 36.7°) appeared with the increase in Zn amount in ZSM-5. In ZnO/HZ5 samples, the Zn content was higher than 2.07%, and in Zn/HZ5-IM samples, the Zn content was higher than 3.10%. This suggests that the different preparation methods and Zn sources have a significant influence on the distribution of Zn species in zeolite.

**Figure 2:**
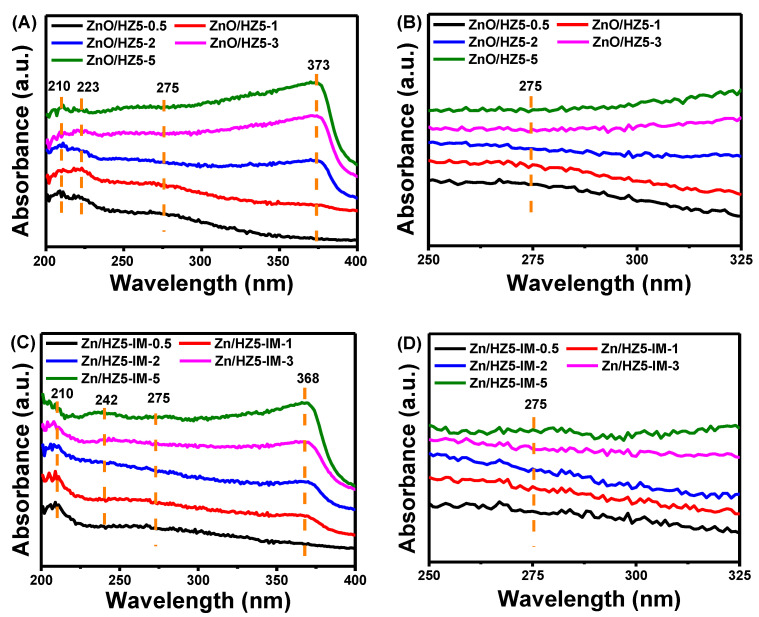
UV-vis spectra of Zn/HZ5 with different Zn sources and Zn content in (**A**) ZnO/HZ5−*x*; (**B**) locally enlarged image of ZnO/HZ5−*x* samples; (**C**) Zn/HZ5-IM−*x*; (**D**) locally enlarged image of Zn/HZ5-IM−*x* samples.

Figure 2 depicts the UV-vis spectra of Zn-containing samples with different Zn sources and Zn content. UV-vis is an effective method to analyze the status of Zn species in HZ5 zeolite. It has been well established that the absorption peaks of ZnO at 368 nm and 275 nm correspond to the presence of large-grain ZnO on the outer surface and nano-ZnO clusters in pores of the zeolite, respectively [40,41]. The absorption peak at wavelengths less than 240 nm could be rationally attributed to the existence of a ZnOH^+^ species that undergoes a charge transfer transition with the zeolite lattice O^2−^ to produce the UV-vis signal [42,43]. Figure 2 suggests that Zn species in zeolite were complex due to the co-existence of different states. It is evident that the absorption peak of large-grain ZnO gradually increased with increasing Zn content, which is consistent with the XRD spectra results. Meanwhile, the peak wavelength of large-grain ZnO for the ZnO/HZ5−*x* (373 nm) was larger than Zn/HZ5−IM−*x* (368 nm) catalysts, and the intensity of the peak attributed to ZnO clusters at 275 nm decreased with the content of Zn species accession, which was caused by the large-grain ZnO enhancement. However, the changes in ZnO clusters and ZnOH^+^ species with variations in the preparation method and Zn concentration were not clear.

**Figure 3:**
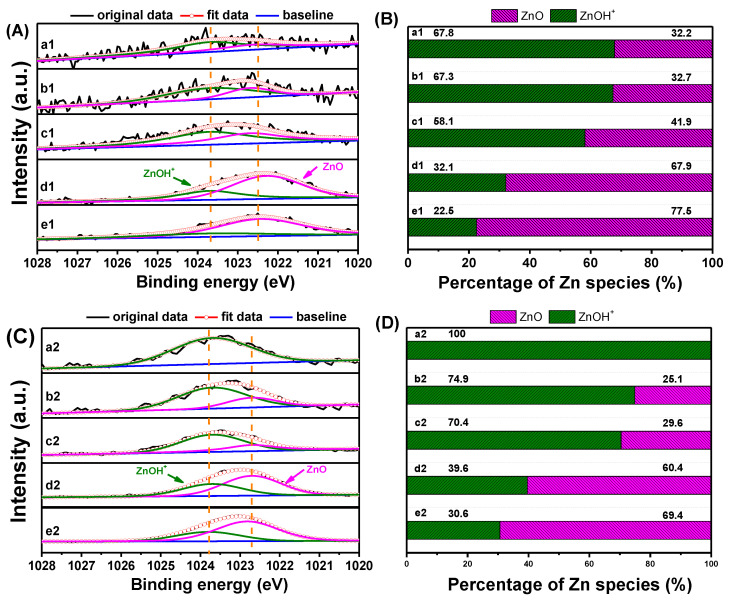
Zn 2p_3/2_ XPS (**A**) and deconvolved results (**B**) of ZnO/HZ5−*x* catalysts, and Zn 2p3/2 XPS (**C**) and deconvolved results (**D**) of ZnO/HZ5-IM−*x* catalysts. a1: ZnO/HZ5−0.5; b1: ZnO/HZ5−1; c1: ZnO/HZ5−2; d1: ZnO/HZ5−3; e1: ZnO/HZ5−5; a2: Zn/HZ5-IM−0.5; b2: Zn/HZ5-IM−1; c2: Zn/HZ5-IM−2; d2: Zn/HZ5-IM−3; e2: Zn/HZ5-IM−5.

The Zn 2p_3/2_ XPS and deconvolved results of ZnO/HZ5−*x* and Zn/HZ5-IM−*x* catalysts are displayed in Figure 3. The shape and position of the XPS spectra indicate that the introduction method and content of zinc species significantly affected the existing state of the surface zinc species. In Zn/HZ5-IM−*x* and ZnO/HZ5−*x* samples, the binding energy decreased with the increase in the zinc content. However, it was clear that the binding energy of Zn 2p_3/2_ in Zn/HZ5-IM−*x* was a little higher than that in ZnO/HZ5−*x* at the same zinc content (Figure 3A,C). Through deconvolution of the XPS spectra, two types of zinc species were identified in the Zn-containing HZSM-5 samples, with binding energies of approximately 1023.7 and 1022.7 eV, corresponding to ZnOH^+^ and ZnO, respectively [44]. It was observed that the content of ZnO species increased with zinc loading, while the content of ZnOH^+^ species decreased. The content of ZnOH^+^ species in the Zn/HZ5-IM−*x* was higher than in the ZnO/HZ5−*x* with the same Zn content, which is consistent with the results obtained from UV-vis and XRD analyses (Figure 3B,D).

### 2.2. Acid Properties

**Figure 4:**
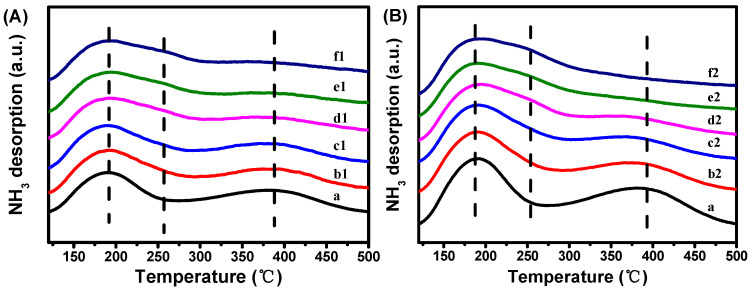
NH_3_-TPD profiles of HZSM-5, (**A**) ZnO/HZ5−*x*, and (**B**) Zn/HZ5-IM−*x*. a: HZSM-5; b1: ZnO/HZ5−0.5; c1: ZnO/HZ5−1; d1: ZnO/HZ5−2; e1: ZnO/HZ5−3; f1: ZnO/HZ5−5; b2: Zn/HZ5-IM−0.5; c2: Zn/HZ5-IM−1; d2: Zn/HZ5-IM−2; e2: Zn/HZ5-IM−3; f2: Zn/HZ5-IM−5.

The concentration and distribution of acid sites in HZSM-5 and Zn-containing HZSM-5 are summarized in Table 2. As shown in Figure 4, the introduction of Zn species had a significant impact on the distribution of acid sites in HZSM-5 zeolites. In HZSM-5, the total amount of acid sites was found to be the highest (0.77 mmol·g^−1^). However, upon the introduction of Zn species, the strength of strong acid sites gradually decreased with increasing Zn content, regardless of the preparation method used. Interestingly, the strength of medium acid sites emerged and increased with the Zn content, implying a synergistic interaction between the Zn species and the inherent acid sites in the parent HZSM-5 zeolite [39]. Furthermore, the methodology for Zn incorporation profoundly influenced the amount of acid sites. Compared to Zn/HZ5-IM−*x*, the ZnO/HZ5−*x* catalysts exhibited a sharp decrease in the amount of acid sites. This can be attributed to the presence of large-grain ZnO, which covers the acid sites.

### 2.3. Analyses of Zn Distribution by EXAFS and LCF of XANES Spectra

It is widely recognized that the Zn species in zeolite are highly complex and may include ZnO particles, ZnO nanocrystals, ZnO clusters, ZnOH^+^, and Zn^2+^ with the structure of O−Zn^2+^−O or [Zn−O−Zn]^2+^ [45,46]. These species with distinct locations, local geometries, and chemical environments result in different catalytic behaviors. Hence, it is crucial to determine the distribution of Zn species. However, the typically highly dispersed state and low concentrations make it challenging to clearly elucidate the nature of Zn species in zeolite. Therefore, EXAFS and XANES techniques, which are effective in looking selectively at a specific chemical constituent and sensitive to the local structure of metal ions, were used to provide the local geometrical information and proportion of Zn species quantitatively by the linear combination fitting (LCF) method.

Several typical Zn compounds were chosen as reference samples to represent potential Zn species that may exist. The identified Zn species in the present work include ZnO particles with a grain size of approximately 50 nm, Zn/HZ5-IE−0.66% representing ZnOH^+^ species, Zn/silicalite-1−0.1% representing confined ZnO clusters in pore channels, Zn/silicalite-1−5% representing crystalline ZnO attached to the outer surface, and Willemite referred to as a Zn−O−Si structure [47,48].

**Figure 5:**
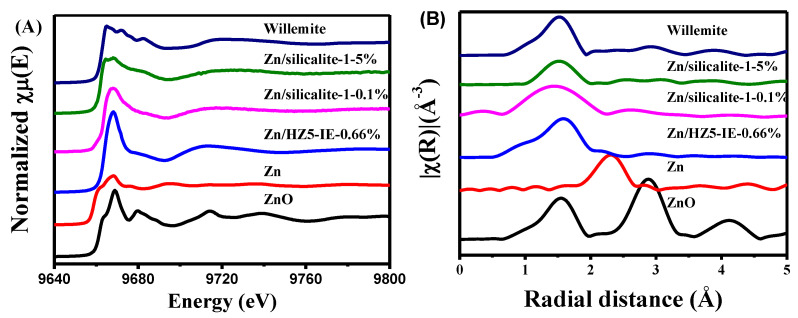
Zn K-edge (**A**) XANES and (**B**) EXAFS spectra of the reference samples.

The background-subtracted and normalized XANES at the Zn K-edge and the plots of the Fourier transforms of the EXAFS spectra (k^2^ weighted over k range from 2.3 to 11.7 Å^−1^) of the reference samples are presented in Figure 5A,B, respectively. The coordination numbers (CN) and coordination distances (R) of oxygen around Zn in each reference sample were calculated by fitting the EXAFS results and are listed in Table 3. The XANES spectra of Zn/HZ5-IE−0.66% and Zn/silicalite-1−0.1% at the Zn K-edge displayed a similar pattern in the white line peak. However, the higher intensity of the white line peak in the Zn/HZ5-IE−0.66% sample suggests a higher oxidation state and coordination number. This is consistent with the E_0_ value and CN data listed in Table 3, which confirms the higher electron density of this sample. The white line peak of the other references, on the other hand, exhibited a different shape with splitting patterns, indicating the presence of different Zn species in each sample.

According to the EXAFS fit parameters of reference samples (Table 3), the first coordination shell at about 1.97 Å for reference ZnO in the radial distribution is attributed to a Zn–O bond, which corresponds to the four nearest oxygen neighbors. With the exception of the Zn/HZ5-IE−0.66% sample, nearly all of the reference samples exhibited a tetrahedrally coordinated Zn species. The differences in radial distance between the Zn and oxygen coordination, as well as the distinct patterns in the white line peaks, indicate the unique local environment of each Zn species. Furthermore, it is noteworthy that the CN of Zn species in Zn/HZ5-IE−0.66% was nearly 6, indicating a sixfold coordinated structure of the Zn atom. This may be attributed to the interaction of the Zn species with the Brönsted acid in the zeolite [49].

**Figure 6:**
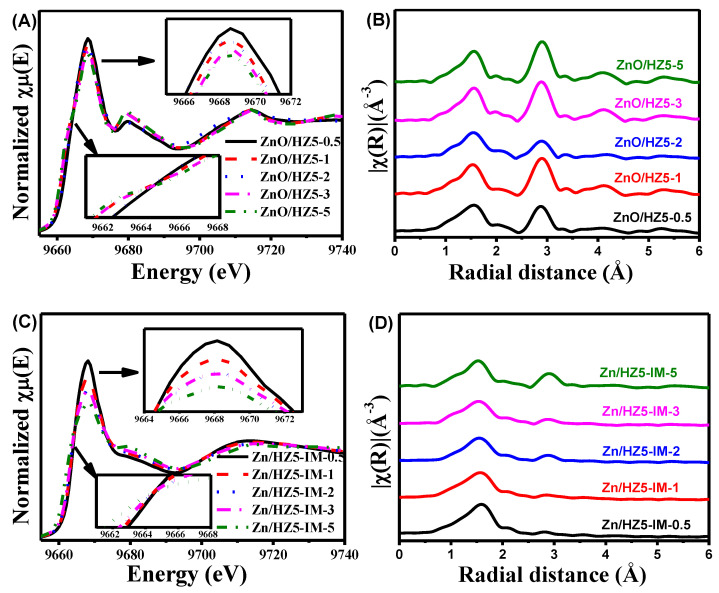
Zn K-edge XANES (**A**,**C**) and EXAFS spectra (**B**,**D**) of ZnO/HZ5−*x* samples (**A**,**B**) and Zn/HZ5-IM−*x* samples (**C**,**D**) with different Zn loadings.

Figure 6 illustrates the Zn K-edge XANES and EXAFS spectra of the Zn-containing ZSM-5 with different Zn contents, and Table 4 summarizes the CN and coordination distances of oxygen around Zn in each sample. In the samples ZnO/HZ5−*x* prepared by the physical mixing method, the white line peak in the XANES spectra display similar patterns but vary in intensity and width (Figure 6A). Notably, the ZnO/HZ5−0.5 sample, which had the lowest Zn content, exhibited the highest absorption edge energy and the strongest oxidation. As the Zn loading increased, the absorption edge (E_0_) shifted towards lower absorption energy, and the intensity of the white line peak decreased, indicating a gradual decline in the oxidation state of Zn species. Meanwhile, the EXAFS spectra and fitted parameters of ZnO/HZ5−*x* (Figure 6B, Table 4) suggest that both the CN and R of oxygen surrounding Zn also decreased from 4.9 to 3.8 and from 2.02 Å to 1.97 Å, respectively, implying the change of Zn species distribution with the content. It is interesting that the second coordination shell from the Zn−Zn bonds in EXAFS spectra (Figure 6B) increased with the Zn concentration. This suggests that Zn species mainly exist in the form of large-grain ZnO species in the physical mixed samples, which is consistent with the results of XRD and UV-vis spectra.

In the samples of Zn/HZ5-IM-*x* prepared using the impregnation method, the change in the white line peak in XANES spectra, as well as the CN and R of Zn-O coordination, with the increase in Zn loading, showed a similar trend to those observed in the ZnO/HZ5−*x* sample (Figure 6C,D, Table 4). Nevertheless, among all the samples investigated, the Zn/HZ5-IM−0.5 sample with the lowest Zn content exhibited the largest CN of 6.2 and R of 2.08 Å. Additionally, the sample showed the absence of a Zn−Zn coordination shell. This suggests that the preparation method had a significant impact on the state of Zn in the samples.

**Figure 7:**
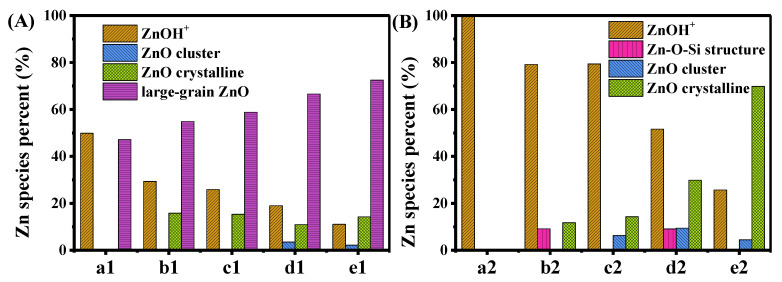
Relative distribution of different Zn species in ZnO/HZ5−*x* samples (**A**) and Zn/HZ5-IM−*x* samples (**B**) with different Zn loadings. a1: ZnO/HZ5−0.5; b1: ZnO/HZ5−1; c1: ZnO/HZ5−2; d1: ZnO/HZ5−3; e1: ZnO/HZ5−5; a2: Zn/HZ5-IM−0.5; b2: Zn/HZ5-IM−1; c2: Zn/HZ5-IM−2; d2: Zn/HZ5-IM−3; e2: Zn/HZ5-IM−5.

A quantitative analysis of the XANES spectra using LCF methodology further determined the proportion of Zn species in various samples (Figure 7). In ZnO/HZ5−0.5 samples, Zn mainly existed in the form of ZnO with large grain size, as well as ZnOH^+^ in ion exchange sites, both of which had similar proportions (Figure 7A). The appearance of Zn ions in ion exchange sites indicated that solid-phase ion exchange occurred during the grinding and 560 °C calcination process in physically mixed samples, which has been reported previously [50,51]. As the amount of Zn loading increased, the relative proportion of ZnOH^+^ decreased, while the content of ZnO with a large grain size increased significantly, and ZnO clusters confined within the zeolite pores were observed. The change in the distribution of Zn species suggests the interaction between different forms of Zn species.

Although only ZnOH^+^ species were observed in the Zn/HZ5-IM−0.5 sample, the change in the distribution of zinc species in the Zn/HZ5-IM-*x* samples showed a similar trend to that in the ZnO/HZ5−*x* samples (Figure 7A,B). This denotes that at low Zn loadings, Zn underwent strong chemical adsorption or direct ion exchange with the Brönsted site of HZ5, resulting in the formation of stable ZnOH^+^ species. In contrast to the samples prepared through physical mixing, the increase in Zn content in the impregnation process resulted in the formation of crystalline ZnO on the external surface of the zeolite, instead of large-grain ZnO that has been obtained by XRD analysis.

### 2.4. Catalytic Performance of Various Catalysts

The ethylene conversion and product distribution found on ZnO/HZ5−*x* and Zn/HZ5-IM−*x* catalysts in the aromatization process are listed in Table 5. It is clear that the distribution of the product varied with the catalysts with different preparation methods and Zn contents. On the catalysts ZnO/HZ5−*x*, the increase in the Zn content resulted in the increase in aromatics from 61.9% to 63.8%, while further increase in the Zn content had a negative effect on the aromatic selectivity. At the same time, the selectivity to H_2_ decreased continuously as the Zn content increased. The catalytic performance of Zn/HZ5-IM−*x* showed a similar trend to that of ZnO/HZ5−*x* with increasing Zn content. However, Zn/HZ5-IM−*x* exhibited a much higher aromatics selectivity compared to ZnO/HZ5−*x* at similar Zn content. This can be explained by the higher concentration of active Zn in Zn/HZ5-IM−*x*.

**Figure 8:**
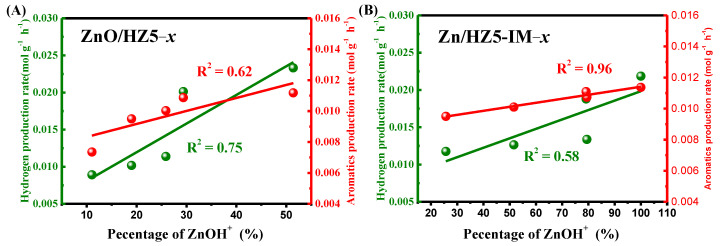
Relationship diagram of H_2_ or aromatics production rate as a function of ZnOH^+^ contents on (**A**) ZnO/HZ5−*x* and (**B**) Zn/HZ5-IM−*x*.

The production rate of H_2_ and aromatics were thus calculated as a function of the proportion of ZnOH^+^ on different Zn-containing HZSM-5 catalysts, as shown in Figure 8A,B. For ZnO/HZ5−*x* and Zn/HZ5-IM−*x* catalysts, there was a linear correlation between the H_2_ formation rate and the proportion of ZnOH^+^ species determined by the LCF method (R^2^ = 0.58~0.75), indicating that ZnOH^+^ species plays a role as active sites for dehydrogenation. Furthermore, a stronger linear correlation between the aromatics formation rate and the proportion of ZnOH^+^ species was observed on Zn/HZ5-IM−*x* (R^2^ = 0.96), confirming the crucial role of ZnOH^+^ in catalyzing aromatics production. However, the situation was different for ZnO/HZ5−*x*, where a weaker correlation was observed (R^2^ = 0.62), indicating the negative impact of large-grain ZnO on the aromatization reaction.

## 3. Experimental

### 3.1. Sample Preparation

The ZSM-5 samples were prepared using the following procedure: a gel system was created by combining silica sol as the source of silicon (40% SiO_2_ aqueous solution, Qingdao Haiwan Specialty Chemicals Co., Ltd., Qingdao, China) as the silicon source, sodium alumina (*x*Na_2_O·Al_2_O_3_, *x* ≥ 1.75, 40% Al_2_O_3_, Sinopharm Chemical Reagent Co., Ltd., Shanghai, China) as the aluminum source, tetrapropylammonium hydroxide (TPAOH, 25% TPAOH aqueous solution, Southwest Institute of Chemical Co., Ltd., Sichuan, China) as the template agent, and deionized water with a molar composition of SiO_2_:0.0167Al_2_O_3_:0.033NaOH:0.15TPAOH:30H_2_O. The mixture was then crystallized at 170 °C for 48 h with rotation. The solid product was then centrifuged, washed, dried overnight at 100 °C, and calcined at 560 °C for 13 h to obtain Na/ZSM-5 zeolite. Next, H-type ZSM-5 zeolite was obtained by ion exchange in NH_4_NO_3_ aqueous solution (1 mol/L, m(liquid)/m(solid) = 50) at 80 °C for 8 h, followed by drying at 110 °C and calcined at 560 °C in air for 8 h. The obtained HZSM-5 sample, referred to as HZ5, was analyzed using ICP-OES and was found to have a Si/Al molar ratio of 28.

ZnO-containing HZSM-5 samples with different Zn contents were prepared by a physical mixing method. In this process, a specific amount of ZnO was mixed and ground with HZ5. The mixture was then calcined at 560 °C for 6 h under an air atmosphere. The resulting samples were labeled as ZnO/HZ5−*x*, where *x* represented the Zn content.

Also, the incipient wet impregnation method was employed to prepare Zn-containing HZSM-5 samples with different Zn contents. A specific amount of Zn(NO_3_)_2_·6H_2_O was dissolved in deionized water, followed by pouring onto the HZ5 zeolite to create a paste. This paste was then left at room temperature for 24 h, followed by overnight drying at 100 °C and subsequently calcining at 560 °C for 6 h. The resulting samples were labeled as Zn/HZ5-IM−*x*, where *x* represents the Zn content.

The preparation of reference samples: Zn foil was provided by the SSRF and BSRF; ZnO sample was purchased from Sinopharm Chemical Reagent Co., Ltd.; Zn/HZ5-IE-0.66% sample was prepared by ion exchanging of HZSM-5 with 0.01 mol/L Zn(NO_3_)_2_ solution (>99% Zn(NO_3_)_2_·6H_2_O, Sinopharm Chemical Reagent Co., Ltd.); Zn/silicalite-1-0.1% sample and Zn/silicalite-1-5% sample were prepared by impregnating silicalite-1 with Zn(NO_3_)_2_ solution with Zn loadings of 0.1 wt.% and 5 wt.%, respectively; and willemite was prepared from hemimorphite (purchased from Jinding Zn mine in Yunnan) by calcination at 1100 °C for 3 h. All the samples were calcined at 560 °C for 3 h before use.

### 3.2. Characteristics of the Catalyst

XRD was characterized by a Rigaku MiniFlex II X-ray powder diffractometer using a Cu Kα ray (λ = 0.154 nm). The tube voltage and current were 30 kV and 15 mA, respectively. The XRD profiles were collected in the 2θ range of 5–40° and a scanning speed of 4°/min.

The content of Zn elements in catalyst was analyzed by an inductively coupled plasma emission spectrometer (ICP-AES, iCAP6300). The surface areas and pore volumes of zeolite samples were measured by N_2_ sorption on a Micrometritics TriStar II 3020 instrument at −196 °C. Prior to the measurement, the samples were dehydrated at 300 °C for 10 h. The total surface area was calculated from the adsorption branch in the range of relative pressure from 0.05 to 0.25 by the Brunauer–Emmett–Teller (BET) method, and the pore volume was calculated from the desorption isotherm by the *t*-Plot method.

A Cary 5000 UV-vis-DRS spectrometer (Agilent Technologies, Santa Clara, CA, USA) was used to collect diffuse UV reflectance spectra. BaSO_4_ was used as a reference sample, and the scanning wavelength was 200–800 nm.

The acid properties of zeolite samples were analyzed by temperature-programmed desorption of NH_3_ (NH_3_-TPD) on a Micrometritics Auto Chem II 2920 chemisorption analyzer equipped with a thermal conductivity detector. Approximately 0.1 g sample was pretreated in He flow (30 mL min^−1^) at 550 °C for 0.5 h and then cooled to 120 °C. Ammonia adsorption onto the zeolite sample was achieved by introducing gaseous NH_3_ into the sample tube until saturation, followed by flushing with He at the same temperature for 2 h to remove physisorbed NH_3_. Then, the TPD profile was recorded from 120 to 500 °C at a heating rate of 10 °C min^−1^. The quantities of strong, medium, and weak acid sites were measured by the amounts of ammonia desorbed at 300–550, 200–300, and 120–200 °C, respectively, through integrating the NH_3_-TPD profile in each temperature interval.

X-ray photoelectron spectroscopy (XPS) was recorded on a Thermo ESCALAB 250 spectrometer with Al Kα radiation source (hν = 1486.6 eV) and a multichannel detector.

X-ray absorption fine spectroscopies (XAFS) were collected at the BL14W1 beam line at the Shanghai Light Source (SSRF) and 1W1B beam line at the Beijing Synchrotron Radiation Facility (BSRF). Zn K-edge XAFS data were collected in transmission mode, using a Si(111) double crystal monochromator for the incident energy scan, and the detection of incident (I_0_) and transmitted (I_1_) photons were used as ionization chambers. A third ionization chamber I_2_ was used for energy calibration and spectra measurement. The storage ring of the SSRF was operated at 3.5 GeV with a maximum current of 300 mA, while the BSRF was operated at 2.5 GeV with a maximum current of 250 mA.

The extended X-ray absorption fine structure (EXAFS) spectra were obtained by using a standard procedure of data reduction and least-square fitting following the IFEFFIT code; the phase and amplitude function were analyzed by using the FEFF 9.0 code. The standard ZnO sample was employed for energy alignment. Data from both the reference and experimental samples were collected within the energy range of 9459 to 10,259 eV. The collected data were then normalized to a unity edge jump using the Athena software from the Demeter package [52]. The χ(k) functions were extracted using the Athena program, and the R-space of the EXAFS spectra were obtained through Fourier transformation. The data fitting was performed in the k-range of 2.3 to 11.7 Å^−1^ and in the R-space range of 1.0–3.5 Å, utilizing a Hanning window and multiple k^n^ weighting (n = 2).

We employed these reference spectra also for a linear combination fit (LCF) analysis of the X-ray absorption near edge spectra (XANES) of Zn/ZSM-5 with different reaction times. LCF analysis was performed in the energy interval 9650–9720 eV, using the Athena code. Each sample XANES, µ^EXP^(E), was fitted as a linear combination of five reference XANES spectra, µ_i_^REF^(E): µ^LCF^(E) = w_1_µ_1_^REF^(E) + w_2_µ_2_^REF^(E) + w_3_µ_3_^REF^(E) + w_4_µ_4_^REF^(E) + w_5_µ_5_^REF^(E). LCF was performed by imposing 0 ≤ w_i_ ≤ 1, but without constraining the sum of the weights to unity, ∑_i_ w_i_ = 1. The best-fit values of ∑_i_ w_i_ were instead examined as an additional indicator of the LCF quality. For each analyzed scan, the corresponding LCF R-factor was computed as ∑_j_ [µ^EXP^_j_(E) − µ^LCF^_j_(E)]^2^/∑_j_ [µ^EXP^_j_(E)]^2^, where j denotes each experimental point in the fitted energy range (9650–9720 eV); R-factor = 0 corresponds to the ideal reproduction of the experimental curve, µ^EXP^(E) = µ^LCF^(E) [53,54].

### 3.3. Catalyst Testing

Ethylene aromatization reaction was performed in a continuous flow fixed-bed reactor (inner diameter of 10 mm, length of 60 cm). A total of 0.5 g zeolite catalyst (20–40 mesh) was pretreated at 470 °C for 12 h in a N_2_ flow (50 mL/min). The reaction was conducted at 470 °C and atmospheric pressure. Ethylene was continuously introduced into the reactor with a weight hourly space velocity (WHSV) of 1.8 h^−1^. The gas products were analyzed online using an Agilent 7890A gas chromatograph (GC) equipped with one thermal conductivity detector (TCD), two flame ionization detectors (FID), and three capillary columns (DB-1, OxyPlot, and Al_2_O_3_/KCl plot). The liquid products were analyzed by an Agilent 7890B GC equipped with a FID, as well as a capillary column (HP-PONA).

The ethylene conversion (*X*) and the product selectivity of hydrocarbons (*S*(*C_i_H_j_*)) and hydrogen (*S*(*H*_2_)) have been defined as

(1) $$C_{2}H4_{\;}\mathrm{conversion}\;(\%):\;X(C_{2}H_{4})=\frac{n\left(C_{2}H_{4},in\right)-n\left(C_{2}H_{4},out\right)}{n\left(C_{2}H_{4},\mathrm{in}\right)}\times 100\%$$

(2) $$\mathrm{Product}\;\mathrm{selectivity}\;(C\;\mathrm{molar}\;\%):\;S\left(C_{i}H_{j}\right)=\frac{i\cdot n\left(C_{i}H_{j}\right)}{\sum i\cdot n\left(C_{i}H_{j}\right)}\times 100\%$$

(3) $$H_{2}\;\mathrm{selectivity}\;(\mathrm{molar}\;\%):\;S\left(H_{2}\right)=\frac{n\left(H_{2}\right)}{\sum n\left(C_{i}H_{j}\right)+n\left(H_{2}\right)}\times 100\%$$

(4) $$H_{2}\;\mathrm{production}\;\mathrm{rate}\;(\mathrm{mol}\;g{-}^{1}\;h{-}^{1}):\;R\left(H_{2}\right)=\frac{S\left(H_{2}\right)\times \sum j\cdot n\left(C_{i}H_{j}\right)}{m\times t}\times 100\%$$

where *n*(*C*_2_*H*_4_, *out*), *n*(*C_i_H_j_*), and *n*(*H*_2_) are the molar amounts of *C*_2_*H*_4_, hydrocarbon product (*C_i_H_j_*) with *i* representing the carbon number in the hydrocarbon molecule, and *H*_2_ discharged from the reactor, respectively, and *n*(*C*_2_*H*_4_, *in*) is the molar amount of *C*_2__4_ fed into the reactor. *m* is the catalyst mass loaded, and *t* is the reaction time.

The molar ratio of *C_i_H_j_*, *n*(*C_i_H_j_*), is calculated from the GC analysis results. In the gas phase, the molar number of *C_i_H_j_* (*i* ≤ 4) is quantitatively determined by combining the molar proportion of *C_i_H_j_* and the total volume of gas flow. In the liquid phase, the molar number of *C_i_H_j_* (*i* ≥ 5) is obtained by calculating the weight proportion of *C_i_H_j_* and the total weight of liquid products.

## 4. Conclusions

The Zn-containing catalysts prepared by different methods and Zn content have both similarities and obvious differences in the distribution of Zn species, no matter which Zn source introduces Zn species. Firstly, the content of ZnOH^+^ species is limited by the degree of ion exchange and shows an initial increase followed by stabilization with increasing amounts of Zn species. Secondly, excessive Zn species tend to aggregate and form ZnO crystallin as well as free large-grain ZnO. This aggregation becomes more pronounced with higher levels of introduced Zn, resulting in XAFS spectrum changes such as a decrease in E_0_ value, a shift in white line peak towards lower energy values, a shortening of bond length, and a gradual decline in coordination number around Zn atoms. When using Zn(NO_3_)_2_ as the source of Zn, XAFS spectra show a higher E_0_ value, bond length, and ZnOH^+^ species, which are also demonstrated by XRD, UV-vis, and XPS.

The species and distribution of Zn are compared by XRD, UV-vis, XPS, and XAFS. Taking ZnO/HZ5−*x* as an example, when the Zn content is less than 2%, XRD characterization fails to identify the characteristic diffraction peak of Zn species; with increased Zn content, XRD can only confirm the existence and evolution of ZnO species, while being unable to distinguish the species of Zn species. Compared with XRD, UV-vis can distinguish the species of Zn, but it cannot give the specific content. XPS offers a substantial advantage over the previous two techniques, as it was more sensitive to the Zn species and content, but only applied to surface characterization and could not represent the bulk phase. On the other hand, XAFS combined with LCF could determine Zn species and distribution and provide a more precise selection for subsequent studies of active species, addressing previously existing limitations in characterization.

It was demonstrated that ZnOH^+^ species exhibits high reactivity, and the rate of H_2_ production in ETA reaction is directly proportional to the percentage of ZnOH^+^ species within the total Zn content. However, ZnO species have the opposite effect due to their own migration, aggregation, and sintering. In addition, the average Zn species value obtained by the LCF method can form a better linear relationship with the activity of catalyst, which will provide a more accurate guide for the study of catalytic active species.

## Figures and Tables

**Table 1:** Textural and physical properties of different samples.

Samples	Crystallinity ^a^ (%)	Zn Content ^b^/ (wt.%)	A_surface_/(m^2^·g^−1^)		Pore Volume/(cm^3^·g^−1^)		
			Total	External	Total	Micro	Meso
HZ5	100	-	373	113	0.36	0.10	0.26
ZnO/HZ5−0.5	97.97	0.52	333	116	0.36	0.11	0.25
ZnO/HZ5−1	96.78	0.81	319	109	0.33	0.10	0.23
ZnO/HZ5−2	97.06	2.07	308	99	0.33	0.10	0.23
ZnO/HZ5−3	89.64	3.77	304	101	0.32	0.09	0.23
ZnO/HZ5−5	80.08	5.49	297	100	0.33	0.09	0.24
Zn/HZ5-IM−0.5	98.23	0.56	350	108	0.33	0.10	0.23
Zn/HZ5-IM−1	94.98	1.04	344	105	0.33	0.10	0.23
Zn/HZ5-IM−2	95.39	1.86	338	100	0.34	0.10	0.24
Zn/HZ5-IM−3	89.01	3.10	341	100	0.32	0.10	0.22
Zn/HZ5-IM−5	84.61	4.99	306	90	0.30	0.09	0.21

^a^: The relative crystallinity was estimated by comparing the total XRD peak area of a zeolite sample in the range of 2θ from 22.5° to 25°, with the parent HZSM-5 having the strongest diffraction intensity. ^b^: Zn content obtained by ICP-OES.

**Table 2:** Acidic properties of different samples.

Samples	Acidity Density (mmol·g^−1^)			
	Weak	Medium	Strong	Total
HZ5	0.39	-	0.38	0.77
ZnO/HZ5−0.5	0.41	-	0.21	0.62
ZnO/HZ5−1	0.44	-	0.20	0.64
ZnO/HZ5−2	0.33	0.08	0.12	0.59
ZnO/HZ5−3	0.24	0.22	0.11	0.57
ZnO/HZ5−5	0.19	0.26	0.12	0.57
Zn/HZ5-IM−0.5	0.44	-	0.24	0.68
Zn/HZ5-IM−1	0.43	-	0.21	0.64
Zn/HZ5-IM−2	0.19	0.25	0.20	0.64
Zn/HZ5-IM−3	0.18	0.24	0.16	0.58
Zn/HZ5-IM−5	0.14	0.20	0.13	0.47

**Table 3:** Zn K-edge XANES and EXAFS fit parameters of reference samples.

Samples	Description	E_0_ (eV)	Zn K-Edge EXAFS Fit Parameters ^a^					
			Contribution	CN	R (Å)	S_0_^2^	σ^2^	R-Factor
ZnO	Large-grain ZnO	9661.7	Zn−O	4	1.97	0.91	0.005	0.001
Zn/HZ5-IE−0.66%	ZnOH^+^	9664.1	Zn−O	5.74	2.07	0.91	0.010	0.005
Zn/silicalite-1−0.1%	ZnO cluster	9663.7	Zn−O	3.77	2.02	0.91	0.007	0.010
Zn/silicalite-1−5%	ZnO crystalline	9662.1	Zn−O	3.90	1.97	0.91	0.007	0.005
Willemite	Zn-O-Si structure	9662.5	Zn−O	3.52	1.95	0.91	0.004	0.020

^a^: CN, coordination number; R: distance between absorber and back scatterer atoms; σ^2^: Debye–Waller factor; R-factor = $\sum_{i}{\left({data}_{i}-{fit}_{i}\right)}^{2}/{\left({data}_{i}\right)}^{2}$; S_0_^2^: the amplitude factor, which was fixed values as a 0.91 form model compound.

**Table 4:** Zn K-edge XANES and EXAFS fit parameters of Zn-containing samples with different Zn contents.

Samples	E_0_ (eV)	Zn K-Edge EXAFS Fit Parameters ^a^					
		Contribution	CN	R (Å)	S_0_^2^	σ^2^	R-Factor
ZnO	9661.7	Zn−O	4.0	1.97	0.91	0.005	0.001
ZnO/HZ5−0.5	9662.6	Zn−O	4.9	2.02	0.91	0.008	0.011
ZnO/HZ5−1.0	9661.9	Zn−O	4.3	1.98	0.91	0.006	0.013
ZnO/HZ5−2.0	9661.7	Zn−O	4.1	2.01	0.91	0.007	0.016
ZnO/HZ5−3.0	9661.4	Zn−O	3.8	1.98	0.91	0.004	0.015
ZnO/HZ5−5.0	9661.2	Zn−O	3.8	1.97	0.91	0.004	0.012
Zn/HZ5-IM−0.5	9664.0	Zn−O	6.2	2.08	0.91	0.009	0.005
Zn/HZ5-IM−1.0	9663.5	Zn−O	5.8	2.06	0.91	0.010	0.004
Zn/HZ5-IM−2.0	9663.2	Zn−O	4.4	2.05	0.91	0.009	0.010
Zn/HZ5-IM−3.0	9662.9	Zn−O	4.6	2.03	0.91	0.010	0.008
Zn/HZ5-IM−5.0	9661.1	Zn−O	3.9	1.98	0.91	0.005	0.005

^a^: CN, coordination number; R: distance between absorber and back scatterer atoms; σ^2^: Debye–Waller factor; R-factor = $\sum_{i}{\left({data}_{i}-{fit}_{i}\right)}^{2}/{\left({data}_{i}\right)}^{2}$; S_0_^2^: the amplitude factor, which was fixed values as 0.91 form model compound.

**Table 5:** C_2_H_4_ conversion and product selectivity on ZnO/HZ5−*x* and Zn/HZ5-IM−*x* ^a^.

Sample	C_2_H_4_ Conv. (%)	Product Selectivity (C Molar %)					H_2_ Selectivity (Molar %)
		CH_4_	C_2_^0^-C_4_^0^	C_3_^=^-C_4_^=^	C_5_ and C_5+_	Aromatics	
ZnO/HZ5−0.5	97.7	5.5	25.3	3.3	4.0	61.9	41.6
ZnO/HZ5−1	98.3	6.3	23.0	2.8	4.1	63.8	39.0
ZnO/HZ5−2	99.6	6.0	28.7	1.3	3.5	60.6	25.5
ZnO/HZ5−3	98.4	5.9	29.6	3.3	2.5	58.7	23.2
ZnO/HZ5−5	98.0	7.4	33.6	0.8	1.0	57.2	21.5
Zn/HZ5-IM−0.5	98.1	5.0	23.9	3.7	2.1	65.4	42.2
Zn/HZ5-IM−1	98.7	6.5	26.0	2.3	1.8	63.4	35.2
Zn/HZ5-IM−2	98.2	5.7	25.5	3.5	3.1	62.2	28.6
Zn/HZ5-IM−3	98.4	5.0	30.6	3.3	1.8	59.3	26.8
Zn/HZ5-IM−5	98.8	4.8	32.3	2.8	1.8	58.3	24.2

^a^: The reaction was carried out at 470 °C and 0.1 MPa with ethylene WHSV of 1.8 h^−1^; the data were acquired at the time on stream (TOS) of 12–24 h.

## Data Availability

Data are contained within the article.
